# Editorial: Socially Situated? Effects of Social and Cultural Context on Language Processing and Learning

**DOI:** 10.3389/fpsyg.2022.855733

**Published:** 2022-03-28

**Authors:** Pia Knoeferle, Ramesh Kumar Mishra, Marcela Peña

**Affiliations:** ^1^Institute of German Studies and Linguistics, Humboldt-Universität zu Berlin, Berlin, Germany; ^2^Berlin School of Mind and Brain, Berlin, Germany; ^3^Einstein Center for Neursciences Berlin, Berlin, Germany; ^4^Center for Neural and Cognitive Sciences, University of Hyderabad, Hyderabad, India; ^5^Cognitive Neuroscience Laboratory, Pontificia Universidad Católica de Chile, Santiago, Chile; ^6^Centro Nacional de Inteligencia Artificial, Santiago, Chile

**Keywords:** social context, language, biases, morality, language prestige, individual differences, affect, aphasia (language)

An increasing number of findings in psycholinguistics, neurolinguistics, and the cognitive sciences suggest that the (non-linguistic) socially interpreted and cultural context can influence language processing and learning. That context could include a speaker's (or bystander's) actions, facial expressions, voice or gaze, and gestures, among others. Given the wide variety of contexts (e.g., real-world, videos, still photographs, drawings, narratives, newspaper texts, poems, movies), and of writers, speakers/comprehenders (of different ages, gender, social status, linguistic, and cultural background), the extent of such social and cultural effects on language processing and learning remains unclear, partially because of the complexity to model their interactions (applying different methodologies). The submissions to this Research Topic help delineate the interplay of the socially interpreted and cultural context for language processing and learning/development.

## 1. Language Development and Learning

A number of contributions to this Research Topic focus on language development and learning and showcase the role of the social and cultural context in this domain. From the contributions we can take away the insight that social context is highly diverse. Modeling its influence on learning likely involves a sophisticated understanding of interdependence between language, the world that an individual perceives and acts in, and characteristics of the individual (e.g., mood and language background).

Pointeau et al. examine how robots learn about causal and temporal event relations. They use a corpus of speech from humans describing simple human-robot interactions. Algorithms are used to extract how function words link events to one another (e.g., statistics on how words like “because”, or “then” link different elements in a situation model). The recovered statistics serve as the input for robot learning (how to interact in question-answering dialogue and how to produce narratives). Other research focuses on language development in infants and with a focus on what extra-linguistic cues like a speaker's gaze can contribute to word learning: Cetincelink et al. review evidence suggesting eye-gaze is important for vocabulary development (also longitudinally), word-object reference, object, and speech processing. One key insight from this review is that word-object mapping benefits from eye-gaze. But it remains to be seen to what extent eye-gaze constitutes a mechanism of enhancing learning even more broadly. In addition to benefits of language-world mapping for infants, second language learners also benefit from (the wider pragmatic) context. Zhiwei Bi used a role-play task involving, for instance, requesting a reference letter from a professor or scheduling a meeting with peers. The goal was to ascertain second language learners' strategies. Qualitative analyses of retrospective verbal reports uncovered a range of language-related strategies for speech acts like requests (e.g., comprehending, linking to prior experiences, or pragmatic awareness). Insights into the neurocognitive basis of language learning in social contexts come from a contribution by Kissler and Bromberek-Dyzman. They compared L1 vs. L2 comprehenders' emotion word processing as a function of mood. Mood induction influenced the very first moments of emotion word processing (stronger left-lateralization of mean amplitude in event-related brain potential negativities around 100 ms, the so-called “N1” for happy compared to sad mood). Regardless of first or second language background, valence modulation in the earliest moments emerged following happy but not sad mood induction; language background did modulate later, lexical-semantic processes. A comprehender's second language can also serve as context for, and influence, the decay of native language (“attrition”). A review by Gallo et al. focuses on how first-language attrition happens, why it occurs, and who attritors are (e.g., immigration history, linguistic behaviors, internal neurocognitive states). The authors argue that looking at attrition within the context of the bilingual mind can improve our understanding of how socio-cultural factors (that likely go hand in hand with immigration and first-language attrition) can modulate linguistic processing.

## 2. Expectations and Biases

Social context not only influences language development and learning but also moment-to-moment language processing. The contributions to this Research Topic convey the insight that social context of different sorts seems to enable the formation of expectations. Expectation-formation has been called into question for lexical-level cues (see DeLong et al., 2017; Ito et al., 2018; Nieuwland et al., 2018; Nieuwland, 2019) and against this backdrop the convergence in anticipatory social context effects is striking. A close look at the results clarifies, however, that anticipation is not the same for all world-language relations. Emergence of biases in expectations is also striking. Guerra et al. provide evidence for expectations by exploring the role of gender attitudes and stereotypes in language comprehension. Using visual-world eye-tracking^1^, they found that participants, when inspecting a display with several images, exploited the verb in German sentences to anticipate a character (out of two) that fit with verb gender-stereotype knowledge. These effects were asymmetric in that they were larger for female than male stereotypes but they did not vary between participants depending on their gender (e.g., sexist) attitudes. Anticipation biases also emerged in research examining “common ground” effects (knowledge shared by a speaker and an addressee). Richter et al. used a referential communication game to examine whether common ground is integrated quickly or with delay, involving effort, and whether what is common knowledge vs. privileged (for just the listener) is integrated at the same time. Objects were shown visually either in privileged or common ground, and for critical trials, common ground was relevant but objects in privileged ground had to be ignored. The results from a range of methods, among them eye-tracking and event-related brain potentials, suggested that common ground had early effects, enabling the anticipation of objects; but conflicting information in privileged ground had the potential to interfere. Maquate and Knoeferle complement these insights into common ground effects with a comparison of how referentially-mediated action depictions and non-referentially mediated emotional cues (speaker face emotion) modulate visual attention and language comprehension. Effects of depicted actions were replicated and were pervasive; speaker face emotion effects were, by contrast, more subtle, highlighting the need to pay attention to the relation between language and the world in deriving predictions of (social) context effects.

## 3. Morality, Language Prestige, and Register

Context takes many facets, including that of morality and prestige. 't Hart et al. examined how facial muscle movements in response to emotionally valenced sentences vary depending on whether a sentence protagonist was described as morally good or bad (more frowning upon reading *Mark is angry* vs. *Mark is happy* when Mark was pitched as a good person, but not when he was characterized as bad). Whether the participant was part of the same group as Mark or not (in-group vs. out-group) did not modulate the frowning of the target expression, *Mark is angry/happy* (more frowning muscle activity emerged in the corrugator supercilii for angry than happy sentences). Being part of a social and age group, did, by contrast, affect the performance of participants in a language task (an implicit association test). Weirich et al. reported implicit association test results that differed for older and younger language users and for multi- vs. mono-ethnic groups. Participants, for instance, classified words (of different language register) as having bad or good valence. Experiment 1 contrasted a standard German with input labeled as a low-register German variety and Experiment 2 used the same stimuli but labeled them as standard German vs. standard French. Results revealed that older language users had a stronger association of low-register words with negative valence words when listening to low-register variety, and a smaller effect (less negative attitude) when listening to a French-native learner of German. A group of younger participants of mono-ethnic origin, by contrast, had no effect of language variety; but the younger multi-ethnic participants linked the low-register variant to negative-valence words more strongly for French than the low-register German language variety. Liu et al. contributed a study on Chinese (examining the identification of written Chinese characters that were either morally positive or negative valenced); they reported faster identification when positive if the characters were oriented upright or facing to the right. By contrast, immoral characters were identified faster when these were distorted or presented with a left rotation. The authors interpreted these results as suggesting that physical cues like the direction of orientation contribute to encoding social concepts in language, in line with Conceptual Metaphor Theory.

## 4. Individual Differences: Attitude, Affect Scores, and Language Background

The attitude participants have toward contextual information also plays an important role in language processing. This insight emerges from the results of Horchak and Vaz Garrido. The authors focused comprehenders' attention on environmental issues (*noticed ... garbage*) compared with on the emotions of protagonists (*got upset with the garbage*) or on actions (*picked up... garbage*) and assessed to what extent such a focus influenced a comprehender's sentence ratings (seriousness of the issue) and verification (fit with the picture), also as a function of participants' environmental awareness. The results suggested that focus on a topic like environmental issues boosted ratings of sentences with environmental focus, and that participants' environmental awareness can modulate attention in sentence processing. Modulation of language processing by participant characteristics was also observed in Dwivedi and Selvanayagam. They replicated increased mean amplitude negativities “N400” to semantically mismatching vs. matching words in a sentence context. Crucially, these effects were modulated by participants' affect score (PANAS). Larger N400 differences emerged for individuals with smaller negative affect scores, further highlighting the role of individual differences. Kissler and Bromberek-Dyzman reported individual differences for lexical-semantic processing as reflected in the N400, too. N400 mean amplitude differences were larger for second-language than first-language comprehenders. Together these findings highlight the role of individual differences as a modulating factor for context effects.

## 5. Clinical Cases

Doedens et al. examined the role of context, and in particular familiarity with a communication partner in collaborative communication of aphasic patients compared with healthy controls. Measures of communicative efficiency like the time it took participants to complete the goal of the communicative task differed when comparing patients with aphasia to the controls. As instructors in the task, the patients were faster with an unfamiliar (vs. familiar) interlocutor (accuracy was unaffected by interlocutor familiarity). Healthy controls had higher accuracy when the partner was unfamiliar but reaction times were unaffected by the familiarity manipulation. In the listener role, patients showed a boost in accuracy for the unfamiliar interlocutor. A better understanding of how contextual factors influence communication in patients is the first step in intervention studies. A contribution that also speaks to this issue comes from Sanchez-Perez et al. who investigated vocabulary in 2 to 4-year-old children who were on the autism spectrum. They examined the children's vocabulary in at-home and pre-school contexts. Results suggest clear differences in vocabulary (size) across these two contexts, meaning that vocabulary size may be underestimated if only one context is considered.

## 6. Summary Statement

Social and cultural context influences language processing and learning during a lifespan with at least some variability across diverse language user groups.

## Author Contributions

PK conceptualized the editorial and provided a first draft and revised it after feedback. RM and MP provided feedback on the draft. All authors contributed to the article and approved the submitted version.

## Funding

We acknowledge that this research was funded by the Deutsche Forschungsgemeinschaft (DFG, German Research Foundation) – SFB 1412, 416591334, Centro Nacional de Inteligencia Artificial CENIA, FB210017, and FINANCIAMIENTO BASAL PARA CENTROS CIENTIFICOS Y TECNOLOGICOS DE EXCELENCIA de ANID to MP.

## Conflict of Interest

The authors declare that the research was conducted in the absence of any commercial or financial relationships that could be construed as a potential conflict of interest.

## Publisher's Note

All claims expressed in this article are solely those of the authors and do not necessarily represent those of their affiliated organizations, or those of the publisher, the editors and the reviewers. Any product that may be evaluated in this article, or claim that may be made by its manufacturer, is not guaranteed or endorsed by the publisher.

## Footnotes

^1^Visual-world eye-tracking is a paradigm in which images are presented together with spoken language; visual interrogation of the images is tracked and has been found to be guided by the interaction of visual context with language (Cooper, 1974; Tanenhaus et al., 1995; Huettig et al., 2011).
